# A novel Bcr-Abl–mTOR–eIF4A axis regulates IRES-mediated translation of LEF-1

**DOI:** 10.1098/rsob.140180

**Published:** 2014-11-12

**Authors:** Becky Pinjou Tsai, Judith Jimenez, Sharon Lim, Kerry D. Fitzgerald, Min Zhang, Charles T. H. Chuah, Haley Axelrod, Luke Nelson, S. Tiong Ong, Bert L. Semler, Marian L. Waterman

**Affiliations:** 1 Department of Microbiology and Molecular Genetics, School of Medicine, University of California, Irvine, CA, USA; 2 Division of Hematology/Oncology, Department of Medicine, School of Medicine, University of California, Irvine, CA, USA; 3 Cancer and Stem Cell Biology Signature Research Program, Duke–NUS Graduate Medical School, Singapore; 4 Department of Haematology, Singapore General Hospital, Singapore; 5 Department of Medical Oncology, National Cancer Centre, Singapore; 6 Department of Medicine, Duke University Medical Center, Durham, NC, USA

**Keywords:** LEF-1, Bcr-Abl, IRES, mTOR, eIF4A, PP242

## Abstract

Internal ribosome entry sites (IRESs) in cellular mRNAs direct expression of growth-promoting factors through an alternative translation mechanism that has yet to be fully defined. Lymphoid enhancer factor-1 (LEF-1), a Wnt-mediating transcription factor important for cell survival and metastasis in cancer, is produced via IRES-directed translation, and its mRNA is frequently upregulated in malignancies, including chronic myeloid leukaemia (CML). In this study, we determined that *LEF1* expression is regulated by Bcr-Abl, the oncogenic protein that drives haematopoietic cell transformation to CML. We have previously shown that the *LEF1* 5′ untranslated region recruits a complex of proteins to its IRES, including the translation initiation factor eIF4A. In this report, we use two small molecule inhibitors, PP242 (dual mTOR (mammalian target of rapamycin) kinase inhibitor) and hippuristanol (eIF4A inhibitor), to define IRES regulation via a Bcr-Abl–mTOR–eIF4A axis in CML cell lines and primary patient leukaemias. We found that *LEF1* and other IRESs are uniquely sensitive to the activities of Bcr-Abl/mTOR. Most notably, we discovered that eIF4A, an RNA helicase, elicits potent non-canonical effects on the *LEF1* IRES. Hippuristanol inhibition of eIF4A stalls translation of IRES mRNA and triggers dissociation from polyribosomes. We propose that a combination drug strategy which targets mTOR and IRES-driven translation disrupts key factors that contribute to growth and proliferation in CML.

## 2. Introduction

### 2.1. Wnt signalling and LEF-1 in leukaemia

The Wnt signalling pathway has been shown to regulate the proliferation, survival and differentiation of haematopoietic cells [1,2]. Lymphoid enhancer factor-1 (LEF-1) is a transcription factor that mediates Wnt signals in haematopoietic cells by recruiting the co-activator β-catenin to activate Wnt target genes [3]. Knockout studies show that the actions of LEF-1 mediate Wnt signals of proliferation and survival in immature haematopoietic cells [4]. In the case of disease, abnormal regulation of Wnt components, such as aberrant expression of LEF-1, have been implicated in solid cancers and leukaemias [3,5–7]. In haematopoiesis, aberrant and constitutive expression of LEF-1 perturbs differentiation by disrupting normal expression of cell cycle and growth-promoting genes, such as *cyclin D1* and *c-MYC* [3]. Recently, LEF-1 expression was shown to be critical for the proliferation and survival of leukaemia cells, and knockdown of LEF-1 in myeloid leukaemia cell lines (K562 and HL-60) resulted in rapid cessation of growth followed by apoptosis [8,9]. A survey of *LEF1* expression in primary myelogenous leukaemias determined that *LEF1* mRNA and other Wnt target genes (*c-MYC*) are increased in the final blast phase (BP) stages of chronic myeloid leukaemia (CML) compared with those from earlier, slower growing chronic phases (CPs) [7]. While the Bcr-Abl oncogenic tyrosine kinase is the root cause of CML, activated Wnt signalling and its components appear to be involved in the transition to BP. We and others have shown that *LEF1* is a direct Wnt target gene, suggesting that the increase in *LEF1* mRNA at this stage may be due to direct transcriptional activation by an aberrant level of Wnt signalling [7,8,10–12]. Here, we demonstrate an additional mode of misregulation. We find that Bcr-Abl regulates *LEF1* expression at the level of protein production through increased activity of the internal ribosome entry site (IRES) in the 5′ untranslated region (UTR) of *LEF1* mRNA. We propose that Bcr-Abl provides proliferative advantages in CML cells by misregulating the translation of *LEF1*, and we further propose that this misregulation extends to the general class of mRNAs that contain IRESs.

### 2.2. IRES-mediated translation

In this report, we present a novel mode of regulating *LEF1* production in CML via an IRES, a specialized RNA element in the *LEF1* message. Many of the known eukaryotic transcripts that are regulated by IRESs code for growth-promoting and anti-apoptotic signals. IRESs mediate an alternative mode of translation through recruitment of IRES trans-acting factors (ITAFs), which include both canonical and non-canonical translation initiation factors [13–15]. Since IRESs use a mechanism which differs from normal cap-dependent translation, we found that *LEF1* and other IRES-mediated transcripts (*c-MYC, BCL-2, RUNX1*) are uniquely sensitive to disruptions in their translation by small molecule inhibitors. Although others have shown that disrupting translation with inhibitors of upstream signal transduction (imatinib for Bcr-Abl, PP242/INK148 for mTOR (mammalian target of rapamycin)) reduces the transformation properties of CML cell lines and patient cells, we propose that hippuristanol inhibition of translation directly targets a special eIF4A dependence of the IRES-containing class of mRNAs [16–18].

### 2.3. Translation and cancer

**Figure 1.**
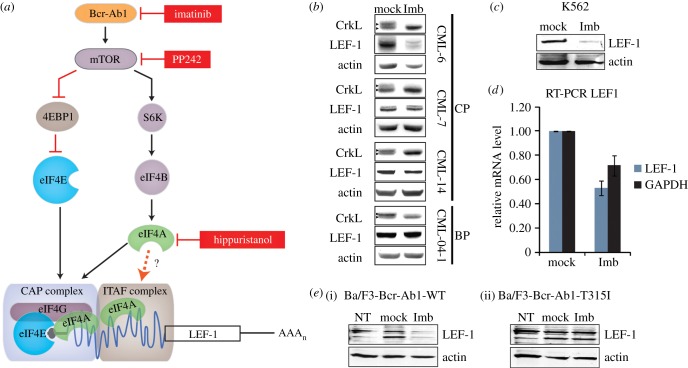
(*a*) Model for IRES-mediated regulation by a Bcr-Abl–mTOR axis. A Bcr-Abl–mTor kinase cascade regulates IRES-mediated translation of LEF-1. Small molecule inhibition of Bcr-Abl (imatinib), mTOR (PP242) and eIF4A (hippuristanol) reduces LEF-1 protein expression and IRES activity. The CML oncogene Bcr-Abl upregulates the mTOR signalling pathway to promote the suppression of the cap-translation initiation inhibitor (4EBP1) and differentially regulates other components of translation (eIF4B, eIF4A). At high concentrations of the inhibitors, both cap-dependent translation and IRES-mediated translation are shut down. However, IRES elements in cellular mRNAs have an increased requirement for the activities of these translation regulators including eIF4A and therefore heightened sensitivity to inhibitors that reduce activities. These factors are functioning canonically in standard cap-dependent translation complexes as well as non-canonically in ITAF complexes to facilitate an IRES-mediated translation mechanism. (*b*) Western blots showing LEF-1 expression in three patients (CML-6, -7, -14) in chronic phase (CP) CML, and one patient (04-1) in blast phase (BP). Cells were treated with DMSO (mock) or 2 µM imatinib (Imb) for 24 h. A polyclonal antibody was used to detect LEF-1. The presence of imatinib inhibited CrkL (right facing triangles) phosphorylation (higher mobility band), indicating the inhibition of Bcr-Abl. (*c*) Western blot analysis of whole cell lysates from K562 cells treated with the same conditions as described above in (*b*). (*d*) RT-PCR analysis of total RNA isolated from K562 cells treated as described above in (*b*). *LEF1* (Pr2 primer) and *GAPDH* ORF primers were used to detect target mRNAs. (*e*) Ba/F3-Bcr-Abl-WT (i) and Ba/F3-Bcr-Abl-T315I (ii) cells were transfected with a LEF-1 expression plasmid containing full-length 5′ and 3′ UTRs; NT, non-transfected control. Cells were treated with the indicated drugs as described in (*b*) for 6 h and probed for the indicated target proteins.

The significance of translational control in cancer progression has come under recent scrutiny since several oncogenic signalling pathways, including the Bcr-Abl pathway and one of its targets, mTOR, have been linked to misregulation of translation (figure 1*a*) [19,20]. In CML and other malignancies, sustained activation of mTOR kinases affects the function of two important regulators of translation, S6 kinase (S6K1) and the initiation factor-binding protein 4E-BP1, which become hyperphosphorylated in an mTOR-dependent manner; as a result, 4E-BP1 is inhibited while S6K1 and cap-dependent translation are activated (figure 1*a*) [21,22]. It has been shown that Bcr-Abl activity drives mTOR activity and thereby promotes formation of canonical cap-dependent translation initiation complexes [23]. Furthermore, the inhibition of mTOR kinases by PP242 or of Bcr-Abl by imatinib leads to CML cell death, revealing the significance of translation regulation in the pathogenesis of leukaemia [18,23]. Increased production of key proliferation and survival proteins can alter the growth pattern of cells, and indeed several genome-wide polysome profiling studies suggest that there are gene-specific protein synthesis pathways that are critical for tumorigenesis [20,24,25]. While much attention has been devoted to the effects of oncogenic signals on cap-dependent translation via mTOR and its downstream target, the translation initiation factor eIF4E, much less is known about mRNAs that are translated via cap-independent, IRES-driven mechanisms. Such mRNAs are particularly relevant to cancer since they encode proteins that regulate cell proliferation, cycling and survival under conditions that predominate in a growing tumour (amino acid starvation, genotoxic stress, hypoxia, apoptosis) [13]. We show that IRES-mediated translation of eukaryotic transcripts is regulated by mTOR signalling and propose that downstream canonical translation initiation factors have alternative functions on IRESs.

We have previously used a small molecule inhibitor, hippuristanol, to demonstrate that the canonical translation initiation factor eIF4A is a *LEF1* ITAF in IRES-mediated translation [26]. Furthermore, in Bcr-Abl-transformed cells, activated S6K1 has been shown to regulate eIF4A activity [27]. Therefore, we tested whether Bcr-Abl regulation of IRES activity is dependent on eIF4A. Our data suggest a model in which Bcr-Abl/mTOR regulates the expression of IRES transcripts through its control of the major translation component, eIF4A. We propose that these canonical translation factors serve non-canonical functions in IRES-mediated translation. Drug ‘cocktails' that combine specific kinase inhibitors (PP242) as well as small molecules (hippuristanol) and their non-canonical actions can target subsets of growth-promoting transcripts regulated by the Bcr-Abl–mTOR–eIF4A axis.

## 3. Material and methods

### 3.1. Plasmids

The dicistronic vector pRstF-LEF1 which contains 1.178 kb of the *LEF1* 5′UTR, pRstF-LEF(1.2), has been described in Jimenez *et al.* [28]. The *LEF1* open reading frame (ORF) construct used to express full-length LEF-1 in Ba/F3 cells, containing 1.2 kb of the 5′UTR, the full 1.2 kb ORF as well as the 1.2 kb 3′UTR, has been described [28]. The dicistronic reporter plasmid pRstF-LEF1 was used to generate the monocistronic hairpin reporter pSTF-LEF1 by removing the upstream Renilla luciferase ORF with NheI and BsaA1 restriction sites. Deletion of the SV40 promoter from the pSTF-LEF1 plasmid results in a 90% decrease in luciferase activity (data not shown), confirming that the great majority of mRNA transcripts produced from this vector contain the full-length *LEF1* IRES. *BCL-2* (1.149 kb) and *RUNX1* (1.573 kb) IRES sequences were synthesized by GENEWIZ and subsequently cloned into the pRstF backbone using the Cold Fusion Cloning Kit (System Biosciences). The Renilla sequences were removed, as previously mentioned with pRstF-LEF1, to create pSTF-BCL2 and pSTF-RUNX1. *HCV* (363 nt) and *EMCV* (711 nt) IRES sequences were cloned into the pRstF backbone. Monocistronic constructs without the upstream hairpins were also constructed: Mono-LEF1, Mono-cMYC (393 bp) and Mono-PV (676 bp). Mono-LEF1 and Mono-PV were created from pRstF-LEF1 and pRstF-PV, respectively, by removing the Renilla ORF and hairpin with Nhe1 and EcoR1 restriction sties. The mono-cMYC IRES reporter was generated by removing the Renilla luciferase ORF with EcoRV and Spe1 from a dicistronic vector (a gift from Dr. Anne Willis, University of Nottingham).

### 3.2. Cell culture and drug treatments

The haematopoietic cell lines human K562, Jurkat, HL-60, and murine Ba/F3-Bcr-Abl-WT and Bcr-Abl-T315 were cultured in RPMI1640 (Mediatech), 1× medium supplemented with 10% fetal bovine serum, 2 mM l-glutamine and 1× Penicillin-Streptomycin Solution (Mediatech). Cells were maintained at 37°C in a humidified atmosphere of 5% CO_2_. At 24 or 48 h prior to collection, K562 cells were treated with DMSO (mock), 50–250 nM hippuristanol (gift from Dr J. Pelletier, McGill University), 250 nM–2.5 µM PP242 (gift from Dr D. Fruman, UC Irvine), 5 nM–3 µM imatinib (LC Laboratories) or a combination of inhibitors in supplemented RPMI medium.

### 3.3. DNA transient transfections and luciferase/β-galactosidase assays

For transfection of plasmids into K562 cells, approximately 2.5 × 10^5^ cells were seeded into 24-well plates. BioT transfection reagent (Bioland) was used to transfect 500 ng of the respective reporter plasmid and 250 ng of a control cytomegalovirus (CMV)–β-galactosidase (β-gal) plasmid. Cells were treated with the indicated drugs for 24 h or indicated times at 37°C. Cell lysates were prepared for luciferase or β-gal assays 24 h post-transfection. Transfections were performed in duplicates, and each experiment was carried out at least three times (standard deviation plotted where applicable). Cells were lysed with 1× passive lysis buffer (Promega) 24 h post-transfection. Cell lysates were assayed for luciferase activities with luciferin substrate (Sigma) using a SIRIUS luminometer (Berthold Detection). β-Gal activities were determined using the Galacton-Plus substrate (ABS) and Accelerator II reagent (ABS). Per cent raw light units (% RLUs) of firefly luciferase or β-gal activity were determined by the ratio of treated over mock light units. For dual luciferase assays, cell lysates were assayed for luciferase activities using the Dual Luciferase Reporter Assay System (Promega). Percentage activity of firefly or Renilla luciferase activity was determined by the ratio of treated over mock RLUs.

### 3.4. RNA isolation and semi-quantitative RT-PCR analysis

Total RNA was isolated from K562 cells using TriZol reagent (Life Technologies). cDNA was generated by the High Capacity cDNA Reverse Transcription Kit (Life Technologies). Quantitation of target transcripts was performed by real-time PCR using the Maxima SYBR Green Master Mix (Thermo Scientific) on the ABI Prism HT7000 Cycler (ABS). RT-PCR primers used are as follows: *LEF1* primer set 1 (Pr1) forward 5′-CCTTGGTGAACGAGTCTGAAATC-3′ and reverse 5′-GAGGTTTGTGCTTGTCTGGC-3′; *LEF1* primer set 2 (Pr2) forward 5′-TATGATTCCCGGTCCTCCTGGTC-3′ and reverse 5′-TGGCTCCTGCTCCTTTCTCTGTTC-3′; *GAPDH* primer forward 5′-TCGACAGTCAGCCGCATCTTCTT-3′ and reverse 5′-GCGCCCAATACGACCAAATCC-3′. First-strand cDNA synthesis was performed using the High Capacity cDNA Reverse Transcription Kit (Life Technologies).

### 3.5. Western blot analysis

Whole cell lysates were harvested with RIPA buffer (supplemented with protease inhibitors (Sigma)), resolved by electrophoresis on 10% SDS-PAGE (polyacrylamide gels), transferred to nitrocellulose membrane and analysed by immunoblotting. Blots were probed with antibodies (1 : 500–1 : 1000) purchased from Santa Cruz Biotechnology (Actin #sc-1616), Genetex (β-tubulin #GTX107175), Cell Signalling (eIF4A #2013, LEF-1 #4777, c-MYC #5605; P-p70S6K #9234, Crkl #3182, P-Crkl #3181, RUNX1/AML1 #4336, P-4EBP-1 #2855). Whole cell lysates (approx. 20 μg) from Ba/F3-Bcr-Abl-WT and Ba/F3-Bcr-Abl-T315I cells (transfected with 10 µg of the LEF-1 expression plasmid by electroporation) were separated by electrophoresis on 10% SDS-PAGE and probed with the indicated antibodies. For figure 1, LEF-1N polyclonal rabbit antisera (antisera generated in house that detects all LEF/TCF proteins) was used at a 1 : 1000 dilution. Immunoreactive bands were visualized by SuperSignal West Dura Chemiluminescent Substrate (Pierce) after incubation with secondary antibody (GE Healthcare Life Sciences). Images were captured by the Syngene GBox1 1.4L System.

### 3.6. Polysome profiling

For polysome profiling, K562 cells were treated with DMSO or 100 nM hippuristanol in DMSO for 3 and 6 h. Prior to harvesting, cells were treated with 100 μg ml^−1^ cycloheximide (CHX) for 10 min at 37°C. Cells were washed in phosphate-buffered saline (PBS) and resuspended in hypotonic lysis buffer (20 mM Tris pH 7.5, 5 mM MgCl_2_, 100 mM KCl, 0.5% NP-40, 0.5 mM β-mercaptoethanol, 40 U ml^–1^ RNase inhibitor, protease inhibitor cocktail (Sigma), 1 mM PMSF, 100 μg ml^−1^ CHX, 100 nM hippuristanol (supplemented in treated samples only)) and incubated on ice for 15 min. The cytoplasmic fraction was extracted by low-speed centrifugation and loaded onto a 10–50% sucrose gradient. Gradients were centrifuged in an SW40 rotor at 35 000 r.p.m. for 2 h. Gradients were fractionated using an Isco Fractionator by piercing the bottom of the tube and chasing the gradient with a 60% sucrose solution (60% sucrose (w/v), 20 mM Tris pH 7.5, 5 mM MgCl_2_, 1 mM KCl, 40 U ml^−1^ RNase inhibitor, protease inhibitor cocktail, 1 mM PMSF). Fractions were collected with concomitant measurement of the OD 254 nm [29]. RNA was isolated and analysed as previously mentioned.

### 3.7. Flow cytometry and cell counting analysis

Cells were treated with hippuristanol, PP242 or both drugs for 24–48 h prior to processing for flow cytometry analysis. Processing for cell cycle assays was performed with the Click-iT EdU flow cytometry assay kit (Invitrogen). Apoptosis was detected using the FITC Annexin V apoptosis detection kit (BD Biosciences). Flow cytometry was performed for both the cell cycle assay and apoptosis assay on an FACS Calibur flow cytometer (BD Biosciences) and analysed with FlowJo software (v. 7.6.1). Cells were counted by a Z3 Coulter System using a size exclusion counting setting (10–30 µm). Data represent triplicate readings with standard error bars.

### 3.8. Patient samples and cell processing

Peripheral blood (PB) samples were obtained with appropriate consent and IRB approval from patients with CML at the University of California, Irvine (S. T. Ong, #IRB 01–59) and patients seen at the Singapore General Hospital (under SingHealth IRB approved protocols). Patient diagnosis and clinical responses are listed in the electronic supplementary material, table S1 [30]. PB mononuclear cells were obtained by centrifugation through Ficoll-Hypaque, washed in PBS and cryopreserved. CD34^+^ cells were selected by immunomagnetic beads (Miltenyi Biotech). To expand CD34^+^-enriched BP CML cells *in vitro*, cells were thawed and grown (overnight) in serum-free StemPro media (Invitrogen) supplemented with 1× nutrient supplements (Invitrogen) and a growth factor cocktail consisting of 200 pg ml^−1^ granulocyte–macrophage colony-stimulating factor (GM-CSF), 1 ng ml^−1^ granulocyte-colony-stimulating factor (G-CSF), 200 pg ml^−1^ stem cell factor (SCF), 50 pg ml^−1^ leukaemia inhibitory factor (LIF), 200 pg ml^−1^ macrophage inflammatory protein-1 alpha (MIP-1α) and 1 ng ml^−1^ interleukin 6 (IL-6). Cells were then subjected to drug treatment for 48 h in the same media. All cytokines were from PeproTech, except for GM-CSF (sargramostim, Immunex) and G-CSF (filgrastim, Amgen).

### 3.9. Colony-forming assay

CD34-enriched BP cells were thawed and allowed to recover overnight in serum-free StemPro media (Invitrogen), supplemented with human growth factors (GM-CSF 200 pg ml^−1^, G-CSF 1 ng ml^−1^, SCF 200 pg ml^−1^, LIF 50 pg ml^−1^, MIP-1α 200 pg ml^−1^ and IL-6 1 ng ml^−1^) and 1× nutrient supplement (Invitrogen). Cells were then subjected to drug treatment for 48 h, harvested, washed and seeded in methylcellulose (H4434, STEMCELL Technologies). Colonies were counted after two weeks.

### 3.10. Apoptosis assay

CD34-enriched BP cells treated with drugs for 48 h were harvested and washed once with ice-cold PBS and centrifuged for 5 min at 500*g* at 4°C. Cell pellets were stained as described in the manufacturer's protocol (Annexin V-FITC; Beckman Coulter). Briefly, the cell pellets were resuspended with 100 μl of ice-cold binding buffer and stained with 10 μl of Annexin V-FITC for 15 min. Prior to flow cytometry analysis, the reaction mix was diluted with 400 μl of binding buffer (containing 0.5 μg ml^−1^ propidium iodide). Stained cells were analysed by flow cytometry within 30 min.

## 4. Results

### 4.1. LEF-1 expression in chronic myeloid leukaemia

It has been shown that *LEF1* transcripts are upregulated in advanced CML [7]; therefore, we determined whether LEF-1 protein is detectable in primary CML isolates and whether expression is due to the actions of the major oncogene Bcr-Abl or its downstream target, mTOR kinase, a central regulator of cap-dependent translation. Primary lysates from three patients in CP CML and one patient in BP were examined for LEF-1 expression via western blot analysis with a polyclonal antibody that detects all forms of LEF-1 protein (figure 1*b*). Prior to harvest, primary CML cells were treated for 24 h with 2 µM imatinib to inhibit the kinase activity of Bcr-Abl. Parallel blots were probed with CrkL antibody to confirm inhibition of Bcr-Abl kinase activity by imatinib. CrkL is a known target of the kinase and loss of the slower migrating phosphoprotein is evident. Blots were re-probed with actin antibody to control for loading. Full-length LEF-1 is frequently expressed as a cluster of polypeptides from 53 to 57 kDa due to alternative splicing in the middle and 3′ ends of the mRNAs [31]. Here, a major isoform of 55 kDa, and sometimes a minor 57 kDa isoform, was expressed in all four primary CML samples. We observed that LEF-1 protein expression correlated with Bcr-Abl kinase activity in one CP CML sample (CML-6), less so in two other CP samples (CML-7 and CML-14) and not at all in the BP CML sample (CML-04-01).

To determine whether the variable sensitivity of LEF-1 expression to imatinib might correlate with clinical outcome, we reviewed the clinical response of the patients to imatinib treatment (electronic supplementary material, table S1) [30]. Interestingly, we found that patient CML-6 had the best clinical response to imatinib and was able to achieve a complete cytogenetic response (CCR) within seven months of initiating therapy; LEF-1 expression was markedly inhibited by imatinib in this cancer. By contrast, patients with poor clinical outcomes had cancers where LEF-1 expression exhibited varying levels of insensitivity to imatinib. Patient CML-7 failed to achieve a CCR and progressed to accelerated phase CML within 2 years of starting imatinib therapy, while patient CML-14 failed to even achieve a complete haematologic response. The patient whose cells exhibited no LEF-1 sensitivity to imatinib, CML-04-01, fared the worst and died with imatinib-resistant myeloid BP disease within three months of providing the sample. These results demonstrate that LEF-1 protein is expressed in all stages of CML and that Bcr-Abl may regulate LEF-1 expression in imatinib-sensitive CML.

### 4.2. Imatinib reduces LEF-1 protein expression

To determine how LEF-1 expression might be regulated by Bcr-Abl, we turned to the K562 CML cell line which expresses an imatinib-sensitive form of Bcr-Abl. Western blot analysis of K562 cells treated with 2 µM imatinib demonstrated significantly downregulated LEF-1 protein levels (figure 1*c*), an outcome similar to that observed with patient sample CML-6 (figure 1*b*). We conclude that K562, which is an imatinib-sensitive CML cell line, is a valid model system to study LEF-1 regulation by Bcr-Abl, since it recapitulates the pattern of drug sensitivities seen with the imatinib-sensitive patient sample.

Decreases in protein levels can be due to regulation at many different steps from transcription to translation. Indeed, our RT-PCR analysis detected only an approximately 25% decrease in *LEF1* mRNA when Bcr-Abl was inhibited (figure 1*d*). The modest reduction in *LEF1* mRNA compared to the near-complete loss of LEF-1 protein suggested that Bcr-Abl might contribute significantly to post-transcriptional regulation of *LEF1*. Therefore to separate pre- and post-transcription regulatory actions of Bcr-Abl, heterologous expression of LEF-1 was established in a pre-B lymphocyte cell system. Ba/F3-Bcr-Abl-WT and Ba/F3-Bcr-Abl-T315I cells are pre-B lymphocyte cell lines modified to express either imatinib-sensitive ‘wild-type’ Bcr-Abl (Ba/F3-Bcr-Abl-WT), or an imatinib-resistant Bcr-Abl mutant (Ba/F3-Bcr-Abl-T315I). We transiently transfected each of these cell lines with a LEF-1 expression plasmid in which a highly active CMV promoter produces full-length *LEF1* mRNA identical in sequence to the 3.6 kb mRNA detected on northern blots (data not shown). Treatment with imatinib in Ba/F3-Bcr-Abl-WT cells downregulated LEF-1 protein to very low levels (figure 1*e*(i)). This striking regulation was not observed in the Ba/F3-Bcr-Abl-T315I cells, which express the imatinib-insensitive Bcr-Abl mutant (figure 1*e*(ii)). Futhermore, imatinib treatments did not have any effect on the levels of actin, demonstrating that Bcr-Abl specifically and strongly regulates LEF-1 expression. That LEF-1 protein was almost completely shut down in the Ba/F3-Bcr-Abl-WT cells transfected with a heterologous expression plasmid suggests that Bcr-Abl regulates LEF-1 expression at a post-transcriptional step.

### 4.3. Imatinib inhibits IRES-mediated translation of LEF-1

**Figure 2.**
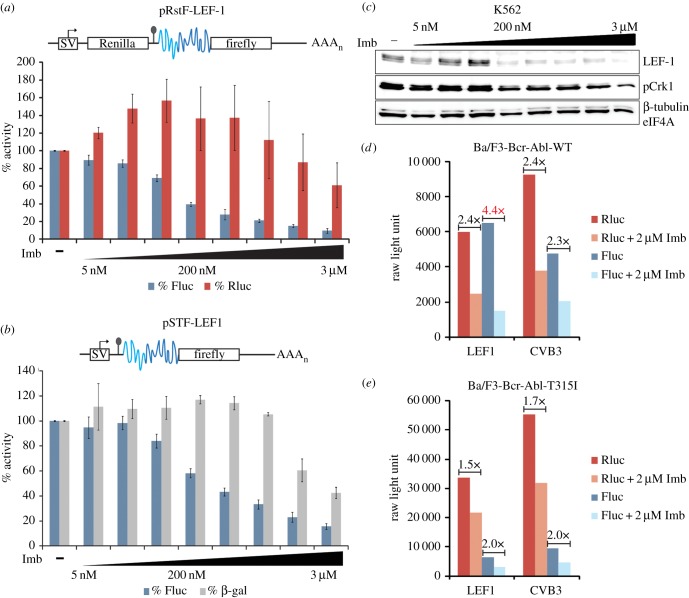
(*a–e*) Bcr-Abl signalling regulates *LEF1* IRES activity. (*a*) Schematic of dicistronic (pRstF-LEF1) IRES reporter vector (IRES region shown in blue) transfected into K562 cells. Cells were treated with titrating concentrations of imatinib (Imb) for 24 h. Luciferase activity is represented as a percentage of activity detected in mock-treated cells. Fluc, firefly luciferase; Rluc, Renilla luciferase. (*b*) Monocistronic hairpin IRES reporter (pSTF-LEF1) transfected into K562 cells and treated with imatinib for 24 h. Activity is represented as a percentage as mentioned above. Fluc represents IRES activity and CMV-β-gal was used as a control. In both IRES reporter constructs, a stem loop sequence (depicted by a line and circle) ensures separate translation of the cap-dependent Rluc ORF from the IRES-driven downstream cistron, Fluc. (*c*) K562 cells were treated with DMSO (–) or titrating concentrations of imatinib (Imb) for 24 h and harvested for western blot. (*d*) Ba/F3-Bcr-Abl-WT cells and (*e*) Ba/F3-Bcr-Abl-T315I cells were transfected with the reporter constructs pRstF-LEF1 or pRstF-CVB3. Cells were treated with DMSO or 2 µM imatinib (Imb) for 24 h. Graph displays RLUs of cap-dependent Renilla (Rluc) and IRES-dependent firefly (Fluc). Fold change of RLUs between DMSO and imatinib-treated samples are displayed above corresponding bars.

In addition to widespread effects on patterns of gene expression, including the transcription of protein biosynthesis genes, Bcr-Abl also directly upregulates cap-dependent translation [21,23]. Thus, in CP and later stages of CML, there is a notable elevation in protein production and cell metabolism [21]. However, LEF-1 protein is not produced by canonical cap-dependent pathways as it uses two IRES modules in its long 5′UTR [28]. While Bcr-Abl regulation of alternative modes of translation is not well defined, positive effects on the IRES-containing *c-Myc* mRNA have been reported [16]. To test whether Bcr-Abl regulates LEF-1 expression through its IRES region, and to definitively distinguish between actions on translation versus other post-transcriptional steps, transient DNA transfections were carried out using both dicistronic and monocistronic hairpin-containing IRES reporter vectors (figure 2*a,b*). Dicistronic assays have been used to demonstrate cap-independent initiation of translation [28]. The dicistronic vector pRstF-LEF1 (figure 2*a*) contains two cistrons separated by an intercistronic region with a hairpin structure [28]. The upstream cistron codes for Renilla luciferase (Rluc) while the downstream cistron encodes firefly luciferase (Fluc). The SV40 promoter (SV) drives transcription of a single dicistronic mRNA with the hairpin structure inserted between the two cistrons to prohibit ribosomal read-through. Using this reporter plasmid design, translation of Rluc relies on cap-dependent ribosome-scanning mechanisms while the downstream Fluc cistron relies on an IRES-mediated process. The dicistronic assay uncouples transcription and translation because the proteins produced, in this case Rluc and Fluc, are generated from a single mRNA. We previously used this assay to demonstrate that translation of *LEF1* is mediated via two IRES modules located in the 5′UTR of *LEF1* mRNA [28]. To mimic an endogenous monocistronic message, we modified the dicistronic IRES reporter by removing the Renilla cistron. This modified reporter, pSTF-LEF1, retains the hairpin structure, which disrupts canonical, cap-dependent translation initiation and eliminates expression of Fluc in the absence of an IRES. To test for Bcr-Abl regulation of IRES activity using these reporter assays, different concentrations of imatinib were tested in K562 cells transiently transfected with either the dicistronic pRstF-LEF1 (figure 2*a*) or monocistronic pSTF-LEF1 (figure 2*b*) IRES reporters. Cells were harvested 24 h post-transfection/treatment, and luciferase assays were carried out to examine *LEF1* IRES activity. Overall, IRES-mediated expression of Fluc decreased with increasing imatinib concentrations (figure 2*a,b*). In addition, the pattern of reduced LEF-1 protein expression closely matched the inhibition of IRES activity and Bcr-Abl activity (as denoted by a reduction in phosphorylation of the Bcr-Abl target protein Crkl; figure 2*c*). Interestingly, IRES activity displayed much greater sensitivity to imatinib (IC_50_ = 150–200 nM) compared to the cap-translated Rluc or β-gal controls (IC_50_ = 2–3 µM).

To test the sensitivity of *LEF1* IRES activity to Bcr-Abl regulation in Ba/F3 cells, reporter plasmid-transfected cultures were treated for 24 h with imatinib and harvested 48 h post-transfection (figure 2*d*). Dual luciferase assays were carried out to examine *LEF1* IRES-mediated translation. The viral IRES of the well-characterized coxsackievirus B3 (CVB3) was included for comparison. IRES (Fluc) and cap-dependent (Rluc) activities are represented as RLUs (figure 2*d,e*). Treatment with imatinib caused a 4.4-fold decrease in *LEF1* IRES activity, while CVB3 IRES activity decreased the same amount as cap-dependent activity (approx. 2.4-fold). While it is possible that Bcr-Abl affects ITAFs commonly employed by viral and cellular IRES elements, these data show that the *LEF1* IRES is more sensitive to the loss of Bcr-Abl activity than the CVB3 IRES or cap-dependent mechanisms (figure 2*d*). As a control, the effects on *LEF1* IRES activity were examined in the imatinib-resistant Ba/F3-Bcr-Abl-T315I cells (figure 2*e*). The results clearly show that increased *LEF1* IRES sensitivity to imatinib is lost in the cell line with imatinib resistance to Bcr-Abl. In the presence of active Bcr-Abl, treatment with imatinib reduced *LEF1* IRES activity more than CVB3 IRES or cap-dependent activity. We conclude that Bcr-Abl regulates *LEF1* expression primarily at the level of IRES-mediated translation.

### 4.4. IRES-mediated translation of LEF-1 is highly sensitive to eIF4A inhibition

**Figure 3.**
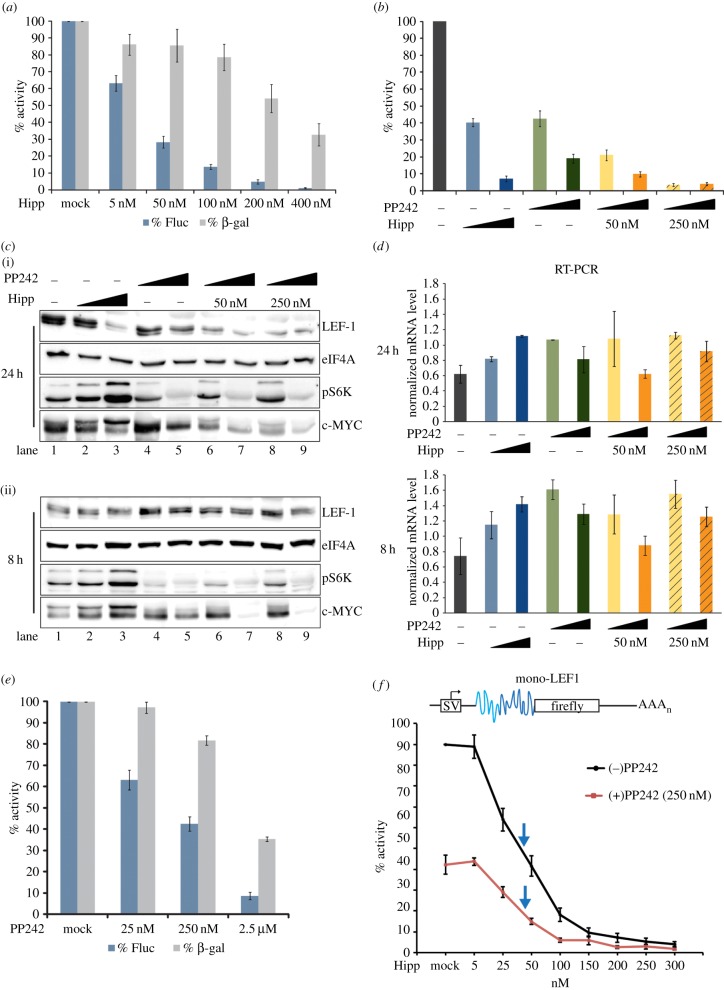
(*a–f*) LEF-1 protein translation is sensitive to the inhibitory actions of hippuristanol and PP242. (*a*) pSTF-LEF1 and β-gal control vector were transfected into K562 cells. Cells were treated with titrating concentrations of hippuristanol (Hipp) for 24 h. Activity is represented as a percentage of the mock-treated cells. (*b*) K562 cells transfected with pSTF-LEF1 and β-gal control vector were treated with DMSO (dark grey), 50 nM (blue) or 250 nM (dark blue) hippuristanol, 250 nM (green) or 2.5 µM (dark green) PP242, or both drugs at alternating low and high concentrations (orange, dark orange). Cells were harvested at 24 h post-treatment for luciferase assays. IRES activity is represented as a percentage of the activity in mock-treated cells. (*c*) K562 cells were treated with hippuristanol, PP242 or both drugs (same concentration as (*b*)) and harvested at 24 h (i) or 8 h (ii) post-treatment for western blot. (*d*) In parallel with protein analysis in (*c*), semi-quantitative RT-PCR was performed to probe for *LEF1* mRNA (Pr2 primers). (*e*) pSTF-LEF1 and β-gal control vector were transfected into K562 cells and treated with titrating concentrations of PP242 for 24 h. Fluc and β-gal activity are represented as a percentage of the activity in mock-treated cells. (*f*) Mono-LEF1 (top schematic) and β-gal control vector were transfected into K562 cells. Cells were treated with titrating concentrations of hippuristanol in the absence (black line) and presence (red line) of 250 nM PP242 for 24 h. IRES activity is presented as a percentage of mock-treated cells. Blue arrows indicate the IC_50_ for each set of drug treatments.

We previously developed the MS2-BioTRAP method to capture and characterize *LEF1* IRES regulatory factors [26]. A specific group of RNA binding proteins was enriched on the *LEF1* IRES (eIF4A, eIF2A, eIF3G, RPL26, YB1, SFPQ, PCBP2, SRp20, hnRNPF), some of which have been previously identified as IRES regulatory factors and others as participants in conventional cap-dependent translation initiation (eIF4A, eIF2A and eIF3G) [14,32]. We focused on eIF4A because a small molecule, hippuristanol, was recently discovered as a potent inhibitor of its RNA binding and helicase activities [33]. eIF4A is a major translation initiation component whose RNA helicase activity unwinds mRNA secondary structure in the 5′UTR to facilitate ribosome recruitment and subsequent formation of the 43S pre-initiation complex [14]. We have previously demonstrated that IRES-driven translation of LEF-1 protein expression is sensitive to hippuristanol inhibition of eIF4A in HEK293 cell lines [26]. These results are consistent with other studies that suggest long and structurally complex 5′UTRs have a strong dependence on eIF4A helicase activity to unwind local structures [34–36]. To address whether eIF4A regulates the *LEF1* IRES in a CML model, we transiently transfected K562 cells with the pSTF-LEF1 and β-gal reporters and treated the cells with increasing concentrations of hippuristanol (figure 3*a,b*, blue bars). The IRES-driven reporter displayed fourfold greater sensitivity towards hippuristanol (IC_50_ = 50 nM) compared with the cap-dependent β-gal reporter control (IC_50_ = 200 nM) (figure 3*a*). Consistent with previous studies on viral IRESs, we confirmed that eIF4A-independent HCV and poliovirus (PV) IRESs displayed greater tolerance for hippuristanol than the eIF4A-dependent EMCV IRES (electronic supplementary material, figure S1*a,b*) [34]. At the level of endogenous proteins, treatment of K562 cells with hippuristanol for 24 h (50 and 250 nM) reduced endogenous LEF-1 protein to 20% of untreated levels and did not significantly alter eIF4A levels (figure 3*c*, lanes 2 and 3). Luciferase assays performed in parallel with endogenous protein analysis showed 60–95% reduction in IRES activity with 50 and 250 nM hippuristanol, respectively (figure 3*b*, blue bars). No change in LEF-1 protein level was observed after an 8 h treatment (figure 3*c*(ii)). Since the half-life of LEF-1 is approximately 13 h, the lack of a change at 8 h suggests an obstruction at the level of translation rather than increased protein turnover [37]. We conclude that LEF-1 protein production is highly sensitive to the activity of eIF4A.

**Figure 4.**
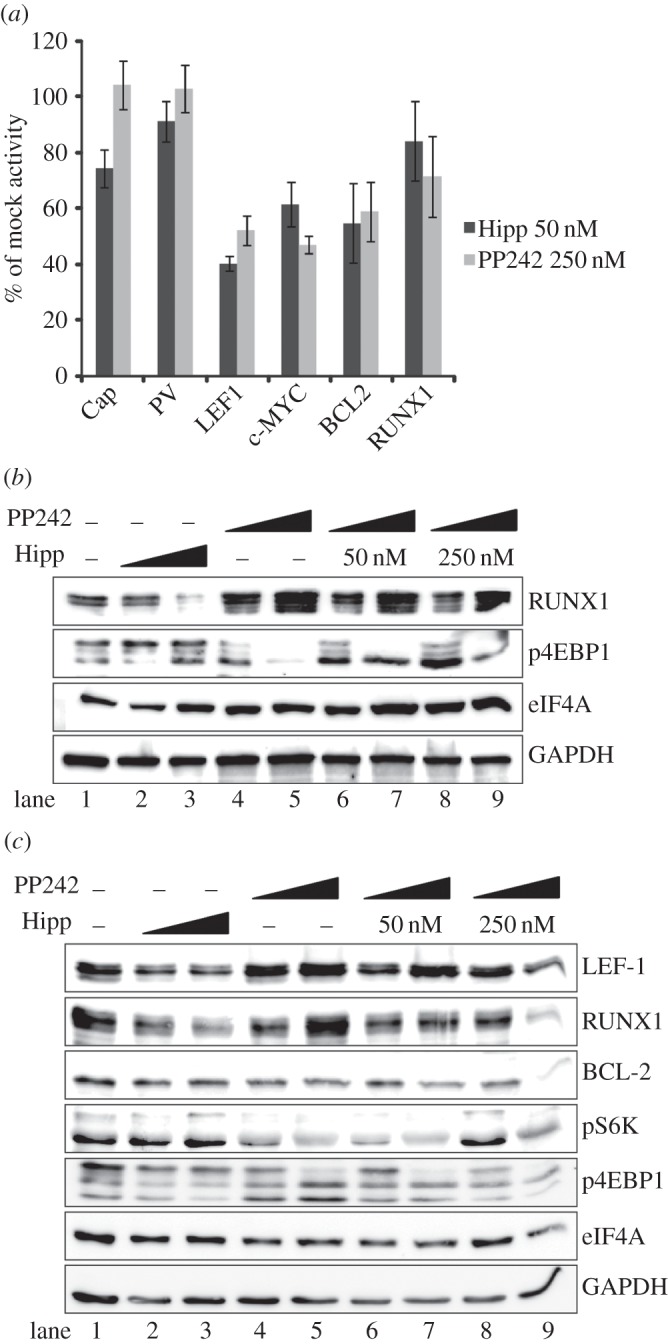
(*a–c*) Hippuristanol and PP242 suppress cellular IRES activity. (*a*) Monocistronic reporters for the cap-translated firefly luciferase (Cap) and the IRESs PV, *LEF1*, *c-MYC*, *BCL2* and *RUNX1*, were co-transfected with the β-gal control vector into K562 cells. Cells were harvested after 24 h with 50 nM hippuristanol (Hipp) and 250 nM of PP242. All raw values were normalized with β-gal and activity is represented as a percentage of the activity of each reporter in mock-treated cells. (*b*) Western blot analyses were performed with K562 cells treated under the same conditions as figure 3*c* and probed with indicated antibodies. (*c*) Jurkat cells were treated with hippuristanol, PP242 or both drugs and harvested at 24 h for western blot analysis.

To confirm that hippuristanol is functioning at the level of translation, we analysed endogenous *LEF1* messages by RT-PCR (figure 3*d*, blue bars). Hippuristanol treatment at both 8 and 24 h showed no reduction in *LEF1* transcripts, and in fact slightly increased its levels. Taken together, the data suggest that IRES-mediated translation of *LEF1* is highly sensitive to the inhibition of eIF4A by hippuristanol. We also observed striking increases in the phosphorylation of c-MYC, another protein produced via IRES-dependent translation (figure 3*c*, lanes 2 and 3). Hippuristanol treatment increased the level of phosphorylated c-MYC (approx. 65 kDa) while decreasing the non-phosphorylated 60 kDa isoform. Although it is not clear how hippuristanol affects c-MYC function, inhibition of eIF4A activity has clear regulatory consequences on phosphorylated c-MYC. We observed two other responses that support eIF4A as a potent and specific regulator of the *LEF1* IRES: (i) hippuristanol increased the levels of phosphorylated S6K, a direct mTORC1 substrate and indicator of active mTOR activity (figure 3*c*, lanes 2 and 3) and (ii) hippuristanol does not change the level of phospho-4EBP1 (the inactive form that cannot inhibit eIF4E/cap directed translation; figure 4*b*, lanes 2 and 3). Hippuristanol induced reduction of LEF-1 protein levels in the presence of increased mTOR and active cap-dependent translation signals support the specific regulation by eIF4A downstream of mTOR/pS6K signalling.

### 4.5. PP242 inhibition of mTOR reduces *LEF1* IRES activity

It has been previously shown that Bcr-Abl drives translation through the mTOR pathway [23]. To investigate whether mTOR signalling is involved in LEF-1 expression, we used the potent dual mTOR inhibitor PP242 to block all mTOR activity. Effective inhibition of mTOR was confirmed by strong reduction of phosphorylated S6K (figure 3*c,* lanes 4 and 5), and under these conditions we observed a readily detectible reduction of LEF-1 protein without any significant changes in *LEF1* mRNA levels (figure 3*d*, green bars). Interestingly, PP242 treatment of K562 cells transfected with pSTF-LEF1 revealed greater sensitivity of the IRES (IC_50_ = 250 nM) than the cap-dependent β-gal control (IC_50_ = 2.5 µM) (figure 3*e*). These data suggest that Bcr-Abl might regulate LEF-1 expression indirectly through the actions of mTOR on *LEF1* IRES factors. To determine whether eIF4A actions on the *LEF1* IRES are downstream of mTOR, we performed a dose–response curve with both hippuristanol and PP242 (figure 3*f*). Cells were transfected with a monocistronic *LEF1* IRES reporter plasmid (mono-LEF1), which lacks the hairpin between the cap and the IRES. These reporters encode authentic 5′UTR structures that resemble an endogenous mRNA, but they nevertheless display the same sensitivity as the previously used pSTF-5UTR (figure 3*b*, green bars; electronic supplementary material, figure S1*d*). Cells were treated with a half maximal dose of PP242 (IC_50_ = 250 nM) in the presence of increasing doses of hippuristanol. Dual treatment further inhibited IRES activity in an additive fashion, and the IC_50_ for hippuristanol remained the same (25–50 nM) with or without PP242. This result suggests that mTOR and eIF4A function within the same pathway for *LEF1* IRES regulation. Western blot analysis revealed that 24 h treatment with both drugs dramatically reduced LEF-1 and c-MYC protein expression (figure 3*c*, lanes 6–9), and since *LEF1* mRNA levels remain relatively unchanged (figure 3*d*, orange bars), the data once again strongly support the notion that LEF-1 protein expression is regulated at a post-transcriptional step via its IRES and that Bcr-Abl regulation of the *LEF1* IRES is mediated by the downstream effectors mTOR and eIF4A.

Although no single signalling pathway has been shown to regulate all eukaryotic IRESs, we asked whether other IRESs could be regulated in the same manner as the *LEF1* IRES. We performed hippuristanol and PP242 drug treatments on three cellular IRESs, (*c-MYC*, *BCL2, RUNX1/AML1*) and one viral IRES (PV), to compare their sensitivity to inhibition of mTOR or eIF4A (figure 4*a*). Interestingly, not all eukaryotic IRESs displayed the same level of sensitivity to a low-dose drug exposure. *LEF1*, *c-MYC* and *BCL-2* showed the greatest sensitivity, whereas the *RUNX1* IRES displayed the least sensitivity (figure 4*a*). In addition, RUNX1 protein levels increased with PP242 treatment (figure 4*b*, lanes 4 and 5), a response which was not reflected in its IRES activity assay, but an effect that rescued hippuristanol inhibition of RUNX1 expression (figure 4*b*, lanes 6–9). Even at higher drug concentrations (250 nM hippuristanol and 2.5 µM PP242), *RUNX1* IRES activity displayed the least sensitivity to both drugs while all other control and IRES-containing transcripts had significantly reduced activity (electronic supplementary material, figure S2). We were unable to monitor BCL-2 in K562 cells as the protein levels were too low for detection.

To address whether the effects on the target proteins were specific to CML or apparent in other mTOR-dependent leukaemias, we tested the Jurkat leukaemia T-cell line. Jurkat cells are deficient in *PTEN* and therefore maintain constitutively active mTOR signalling [38]. Similar to K562 cells, hippuristanol treatment of Jurkat cells reduced both LEF-1 and RUNX1 expression (figure 4*c*). PP242 treatment alone displayed mild or no effects on LEF-1, RUNX1 or BCL-2, but dual drug treatment at the highest concentration decreased the protein levels for all three targets. These experiments highlight differential responses and sensitivity of IRES-containing transcripts to mTOR and eIF4A inhibition in different cellular contexts. Overall, hippuristanol demonstrated the most potent and consistent inhibition of the IRES-containing transcripts (*LEF1*, *RUNX1*) in two different leukaemia cell lines. The mild response to single PP242 treatment of Jurkat cells, but more dramatic response to dual drug treatment, suggests that multiple signals in the mTOR pathway regulate IRES-mediated translation and that a combinatorial drug approach is necessary to significantly reduce protein synthesis. Selective restriction or inhibition of a subset of mRNAs, as shown in figures 3 and 4, suggests a similar mechanism of translation regulation among IRESs in CML and other leukaemias.

### 4.6. eIF4A promotes *LEF1* IRES retention on ribosomes

**Figure 5.**
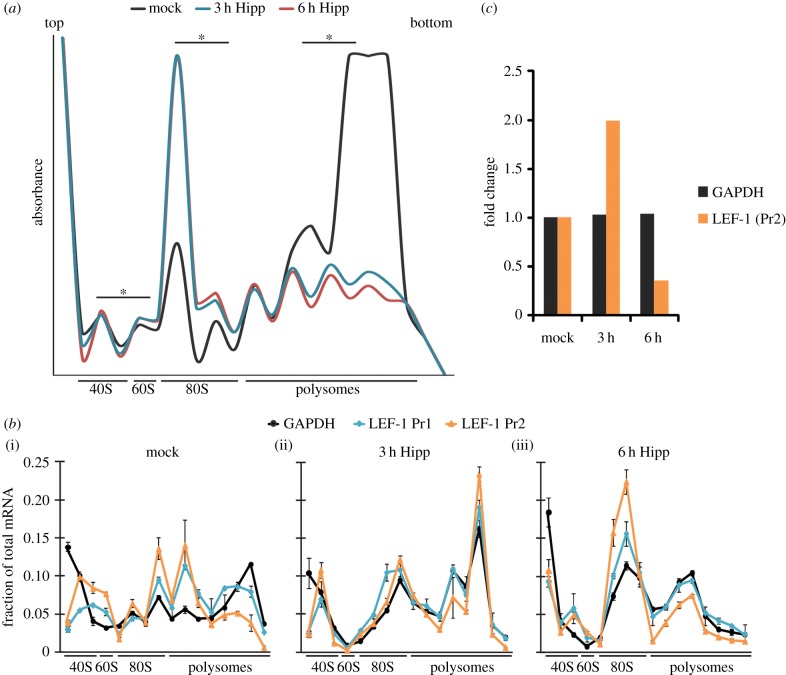
(*a,b*) Polysome profiles of hippuristanol-treated K562 cells. (*a*) Polysome profiling was performed on K562 cells treated with DMSO (mock, black) or 100 nM hippuristanol in DMSO (Hipp) for 3 h (blue) or 6 h (red). The scale of the profile was adjusted for optimal visualization of smaller peaks, resulting in polysome peaks that are off-scale and therefore not displayed. Fractions used to determine the relative quantity of *LEF1* and *GAPDH* mRNA for (*c*) are marked (asterisks). (*b*) RT-PCR of *GAPDH* (black) and *LEF1* (blue, orange) mRNA was performed on total RNA collected from each ribosome population from (*a*). (i) Mock, (ii) 3 h and (iii) 6 h profiles display the fraction of target mRNAs relative to the total mRNA isolate for each ribosome fraction collected. Two sets of *LEF1* primers, Pr1 and Pr2, were used to detect the N-terminal domain (blue) and C-terminal domain (orange), respectively. (*c*) Graph displays the fold change of total *LEF1* and *GAPDH* mRNA (from selected fractions of each ribosome pool marked in (*a*)) from RT-PCR analysis. Fold change of target mRNAs are relative to their corresponding mock-treated samples at 3 or 6 h of hippuristanol treatment. *LEF1* was detected by primer Pr2.

The heightened sensitivity of cellular IRES-driven mRNAs to inhibition of eIF4A or its upstream regulators (Bcr-Abl/mTOR) suggests that eIF4A provides a unique role in IRES-mediated translation initiation compared to its role in cap-dependent translation initiation. Since polyribosome profiling can detect important differences in the association with ribosome subunit joining and translation initiation, we tracked the levels of *LEF1* mRNA in polysomes in the presence and the absence of hippuristanol (figure 5*a*). Hippuristanol treatment disrupted overall polysome assembly at 3 and 6 h as previously reported [33] (figure 5*a*). Within 3 h, we observed a concordant dramatic increase in 80S monosomes, a pattern that reflects inhibition of translation initiation [39]. To examine the functional consequences of hippuristanol treatment on *LEF1* mRNA, we used RT-PCR to determine the distribution of *LEF1* mRNA in the polysomes. In mock-treated K562 cells, both *LEF1* (primer set 1 and 2) and *GAPDH* (control) mRNAs were detected in the polysome fractions (figure 5*b*(i)). Three hours of hippuristanol treatment triggered accumulation of ‘stalled’ or ‘incompetent’ single 80S complexes for both mRNAs (figure 5*b*(ii)). Other peaks in the polysome fractions are likely to derive from elongation competent 80S ribosomes that had already engaged in elongation prior to drug treatment. At 6 h, hippuristanol produced a large peak of *LEF1* and *GAPDH* mRNA accumulation in the 80S fraction (figure 5*b*(iii)) which corresponds to the accumulation of 80S monosomes in the polysome profile (figure 5*a*). Overall, the peaks in the 80S fraction indicate that the loss of eIF4A activity hinders ‘competent’ 80S formation in both IRES and cap-translated transcripts. We showed previously that total cellular *LEF1* mRNA is not reduced in the presence of hippuristanol (figure 3*d*, blue bars). However, the relative level of *LEF1* mRNA associated with 40S and 80S complexes was dramatically reduced by 6 h of treatment compared with *GAPDH* mRNA, indicating a specific loss of *LEF1* transcripts from polysomes and monosomes (figure 5*c*). These results suggest that hippuristanol inhibition of eIF4A disrupts IRES-mediated translation at steps of late translation initiation or early elongation and leads to a sharp disengagement from ribosomes within a few hours.

### 4.7. Hippuristanol and PP242 induce cell cycle arrest in leukaemias

**Figure 6.**
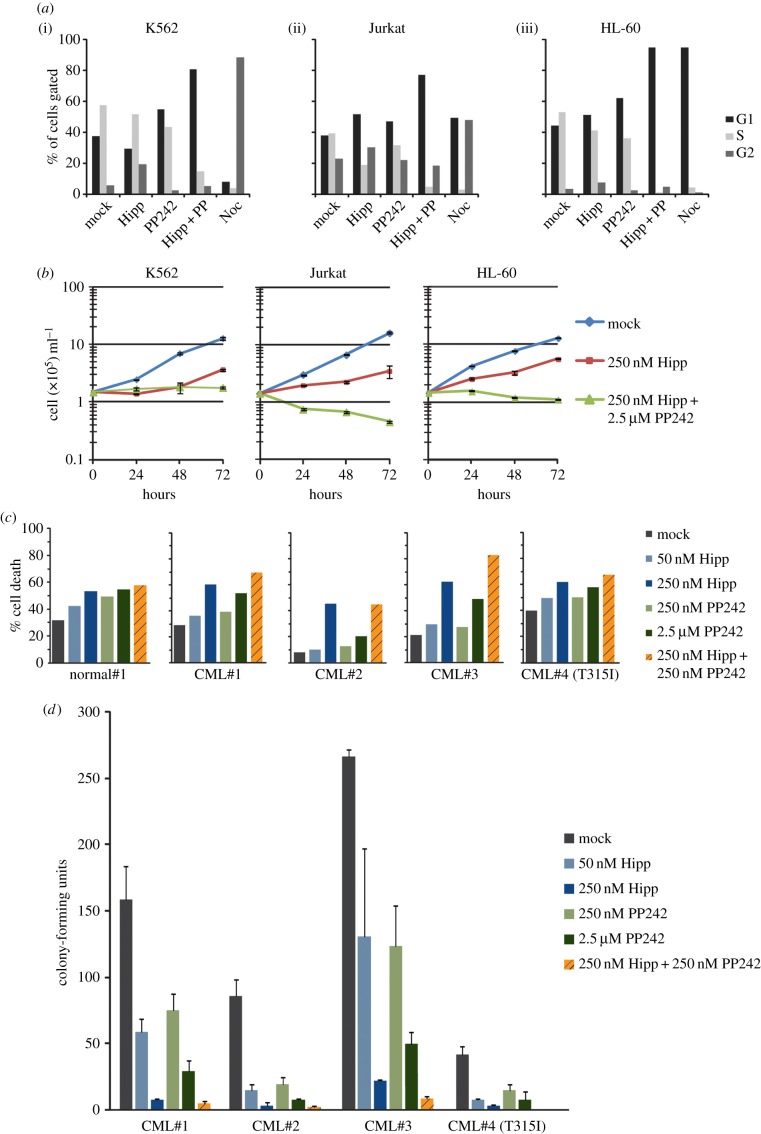
(*a–d*) Effects of hippuristanol and PP242 treatment in leukaemia cell proliferation and survival. (*a*) Cell cycle analysis was performed for (i) K562, (ii) Jurkat and (iii) HL-60 cells. Cells were treated with the following conditions: mock, 250 nM hippuristanol (Hipp), 250 nM PP242, both (Hipp + PP) or 0.1 μg ml^–1^ nocodazole (Noc) for 24 h and analysed by flow cytometry for DNA content. The percentage of cell population in each cell cycle state (G1, S and G2) is indicated. (*b*) Cells counts were determined after 24, 48 and 72 h of drug treatments. (*c*) Apoptosis assay was performed on primary CML patient samples. Cells were treated with hippuristanol, PP242 or both drugs for 48 h, stained with Annexin V-FITC and analysed by flow cytometry. (*d*) Clonogenic assays of primary CML samples after 48 h of drug treatment.

Given the evidence for an mTOR–eIF4A–IRES pathway, we tested how hippuristanol and PP242 influenced cancer cell cycle, cell death and cell proliferation. For cell cycle analysis, we measured DNA content by EdU labelling and flow cytometric analysis for K562, Jurkat and HL-60 (acute myeloid leukaemia, AML) cells (figure 6*a*). In K562 cells, single drug treatment with hippuristanol caused a partial G2 stall (6–19%), whereas PP242 treatment caused a partial G1 stall (37–55%). Dual drug treatment produced a synergistic effect, stalling 81% of the cells in the G1 phase. In Jurkat cells, single hippuristanol or PP242 treatments caused modest stalls in G1 (38–52% and 67%, respectively) and dual drug treatment produced a more pronounced G1 stall (77%). Lastly, in HL-60 cells hippuristanol or PP242 caused modest stalls in G1 (34–50%) or G2 (3–8%), and PP242 also caused modest stalls in G1 (34–61%). However, as seen in K562 cells, dual drug treatment almost entirely stalled the HL-60 cell population in G1 (92%). Although single drug treatments produced minor cell cycle stalls in the G1 or G2 stages, the use of both hippuristanol and PP242 dramatically arrests the majority of the leukaemia cells in G1. These results highlight the important role that mTORC1/2 and eIF4A play in the growth and cell cycle progression of K562 cells and potentially other leukaemias. Analysis of cell numbers over the course of 72 h verified that drug treatments obstructed cell proliferation (figure 6*b*). All three cell lines exposed to dual drug treatment had significantly reduced cell proliferation compared with untreated controls. Apoptosis assays with single and dual drug treatments of K562 cell lines did not show any increase in an apoptotic cell population, indicating that the reduction in cell number was not likely to be caused by drug-induced apoptosis (electronic supplementary material, figure S3). However, the varied apoptotic response from Jurkat and HL-60 cells suggests that the actions of the drugs are context dependent (electronic supplementary material, figure S3). Almost all the primary CML patient cells tested, except for the T315I mutant case, displayed increased cell death with hippuristanol or dual drug treatment compared to cells from a normal control (figure 6*c*). Furthermore, single and dual drug treatments reduced the ability for colony formation for all primary patient CML samples (figure 6*d*). In primary patient CML#2, the level of LEF-1 reduction correlated with the increases in cell death and decrease in colony formation (electronic supplementary material, figure S4; figure 6*c,d*). Our data suggest that subsets of mTOR-dependent cancers are highly susceptible to the loss of mTOR components, where disruption of multiple key factors in the pathway leads to a significant reduction in the capacity for cell growth and proliferation in both leukaemic cell lines and primary CML patient samples.

## 5. Discussion

LEF-1 is a transcription factor that mediates Wnt signalling, and in haematopoietic cells it functions to promote proliferation and survival of differentiating progenitors [4]. Here we demonstrated that LEF-1 is a sensitive target of Bcr-Abl regulation in transformed myeloid cells. We found that this regulation is focused on LEF-1 translation through increased IRES activity. *LEF1* transcripts are known to be upregulated in late stages of blast crisis CML, but to date, translation of this mRNA had not been examined. We have shown that LEF-1 is a target of Bcr-Abl since production of *LEF1* mRNA and LEF-1 protein are downregulated by the Bcr-Abl inhibitor, imatinib. Could LEF-1 play a role in the pathogenesis of CML? Such a role is certainly possible, because the LEF-1 protein is capable of transforming cells [6,40], and we find that it is uniformly expressed in all primary CML samples tested. Furthermore, the degree to which LEF-1 expression could be inhibited by imatinib *in vitro* appeared to correlate with clinical outcome, since lack of inhibition of LEF-1 expression was evident in patient samples with clinical imatinib-resistance (electronic supplementary material, figure S4). These observations suggest that LEF-1 might play a role in drug resistance. Of the known mechanisms associated with clinical imatinib resistance, both Abl kinase domain mutations and Bcr-Abl independent mechanisms appear to be important in the later phases of disease progression [41,42]. Our study strongly supports combination therapy strategies for treating leukaemias—bypassing imatinib resistance by targeting both Bcr-Abl substrates and specific subsets of oncogenic transcripts. A test of these hypotheses will require systematic analysis of a larger group of patient samples.

We used the broad-acting mTOR kinase inhibitor PP242 to determine whether any regulatory target of mTOR was important for LEF-1 expression (figure 3). PP242 and its clinical analogue INK128 have been shown to effectively shut down mTOR signalling, and as a result, destroy leukaemia cells dependent on this pathway [18,43]. We observed that PP242 reduced LEF-1 protein levels, and that low doses combined with a low dose of the eIF4A inhibitor hippuristanol almost completely eliminated LEF-1 expression within 24 h. Therefore, although direct evidence of mTOR/eIF4E regulation of IRES-mediated translation is lacking, our results suggest that IRESs might be highly sensitive to mTOR/eIF4A activities. Recent data from Frost *et al*. [44] also demonstrated that mTOR inhibitors can regulate IRES activity (*VEGF, p27*) and mediate tumour cell apoptosis, underscoring the importance of IRES-mediated translation in response to mTOR signalling in a tumour environment. We propose that in addition to its strong effects on cap-dependent translation, Bcr-Abl/mTOR is an important oncogenic regulator of IRES-directed translation.

Despite the clear connection between aberrant regulation of the canonical translation initation factor eIF4E and tumorigenesis, its oncogenic mechanism is not simply attributed to a general increase in translation activity. In fact, a growing body of evidence suggests that dysregulation of eIF4E and other canonical translation factors such as eIF4A differentially target specific subsets of cancer-promoting mRNAs [35,45,46]. Although IRES-mediated translation of cellular and viral IRESs has been considered cap-independent, our data and findings from other groups suggest that cellular IRESs show some dependence on cap factor eIF4E as well as eIF4A for the formation of the eIF4F initiation complex [14,17,34,47]. Here we show that *LEF1* translation is not solely dependent on eIF4E, but heavily dependent on eIF4A. This distinction derives from our data showing that LEF-1 protein levels are strongly reduced by hippuristanol despite the reactive increase in mTOR signalling (phospho-S6K) and the presence of inactive phospho-4EBP1 and presumably active eIF4E (figures 3*c* and 4*b*). In addition, PP242 inactivation of mTOR and eIF4E does not reduce LEF-1 levels as significantly as hippuristanol inhibition of eIF4A. In fact, both LEF-1 and RUNX1 protein levels *increased* with PP242 treatment in Jurkat cells (figure 4*c*). We speculate that cells are sensitive to disturbances of the mTOR signalling pathway and, thus, treatment with the mTOR inhibitors imatinib or PP242 activates compensatory feedback mechanisms which contribute to varying outcomes. Hippuristanol also activates a positive feedback signal to mTOR, indicating that mTOR senses the reduction of downstream translation components and compensates for this loss by increasing the levels of phospho-S6K. However, even though hippuristanol triggered an increase in the level of phospho-S6K (active mTOR), there was no compensatory increase in LEF-1 or RUNX1 protein synthesis or cap-dependent translation (GAPDH) in CML, Jurkat and AML cell lines (figures 3*c* and 4*b*,*c*; electronic supplementary material, figure S5). The sensitivity of IRES-driven translation (*LEF1*, *RUNX1*) to hippuristanol, in the context of active mTOR, strongly suggests a unique role for eIF4A in the IRES mechanism.

We and others have proposed that long and structurally complex 5′UTRs, such as those observed in IRESs, create mRNAs that are ‘weak’ for translation. That is, these mRNAs may require increased levels of canonical translation factors to overcome rate-limiting steps in translation imposed by IRES structures [14,22,48]. Under normal conditions, IRESs may support ‘low levels' of canonical translation initiation, but are primed to rapidly respond to physiological conditions (cellular stress) that promote upregulation or a ‘switch’ to IRES translation through changes in the availability of ITAFs [32,49,50]. However, in a cancer environment, IRES regulatory factors and signals are upregulated by oncogenic pathways or chronically activated stress signals, and therefore they contribute to the overexpression of IRES-containing mRNAs—many of which code for growth-promoting and survival factors [13,14]. Although the complexity of IRESs and their modes of action differ, we hypothesize that a subset may be regulated in a similar manner in cancer. We tested this idea by inhibiting the primary translation helicase, eIF4A, with hippuristanol. We observed that not all mRNAs are equal in their eIF4A requirements, and that some IRESs have unique dependencies on a subset of canonical translation factors and ITAFs [46]. Several studies have suggested that complex 5′UTRs have an increased need for helicase activity to melt secondary structures and promote formation of the translation initiation complex [34,35,51]. Our results clearly demonstrate that eIF4A inhibition by hippuristanol reduced the activity of multiple eukaryotic IRESs at concentrations that only mildly affect canonical cap-dependent translation (figure 4*a*; electronic supplementary material, figure S1). Furthermore, the reduction of *LEF1* mRNA in ribosome fractions with hippuristanol treatment suggests a possible mechanism where eIF4A is required for ribosome retention of IRES-containing transcripts (figure 5*c*). This sensitivity to the loss of eIF4A activity may suggest a biological role for IRESs as sensors of perturbations in the translation machinery [13,14,52].

To explore the potential of hippuristanol and PP242 as a chemotherapeutic agent, we tested for effects of dual drug treatment on cell survival. Interestingly, neither single nor dual drug treatments induced apoptosis in K562 cells, but induced apoptosis in all primary CML patient samples. Clear reductions in cell proliferation in both cell lines and primary CML cells occured at the highest concentration of both drugs when treated together. Through cell cycle analysis, we determined that the reduction in proliferation was attributed to a striking percentage of cells arrested in G1 (74–94%). Previous reports have noted that PP242 inhibition of mTOR may not be sufficient to reduce cell growth of cancer cells because of its ability to trigger compensating signals that activate the AKT pathway [53]. Our data strongly suggest that hippuristanol targets a critical subset of growth-promoting transcripts, and in combination with PP242 it can shut down the growth of leukaemic cells. Thus, we propose that the combination of these two inhibitors is a new approach to consider for the treatment of leukaemia.

## Note added in proof

During the revision of this manuscript, Wolfe, AL *et al.* 2014. *Nature* **513**, 65–70, reported that a subset of growth promoting mRNAs are regulated by eIF4A in leukemia cells.

## Supplementary Material

Supplementary Tables and Figures
