# Supplementary material for: A novel Bcr-Abl–mTOR–eIF4A axis regulates IRES-mediated translation of LEF-1
Source: Open Biol. 2014 Nov 12;4(11):140180. doi: 10.1098/rsob.140180 (PMC4248067; doi:10.1098/rsob.140180)
Supplement: Supplementary Tables and Figures [file rsob140180supp1.pdf]

Table 1. Patient Clinical Response [30]

	Phase	ELN Response	Comments
**CML-6**	Chronic phase	Optimal response	Complete cytogenetic response (CCyR) occurred within 7 months of starting imatinib.
**CML-7**	Chronic phase	Failure	Failed imatinib treatment, developed E453K mutation, but subsequently experienced an optimal response to dasatinib. This sample was taken at the initial presentation.
**CML-14**	Chronic phase		No response data on this patient.
**CML 04-01**	Blast phase		Imatinib resistant
**CML1**	Blast phase		Imatinib resistant
**CML2**	Blast phase		Patient presented in blast phase. Had CCyR to dasatinib after one month; then lost to follow up.
**CML3**	Blast phase		Imatinib resistant
**CML4**	Blast phase		Imatinib resistant

All blast phase samples were obtained when patients were in blast phase.

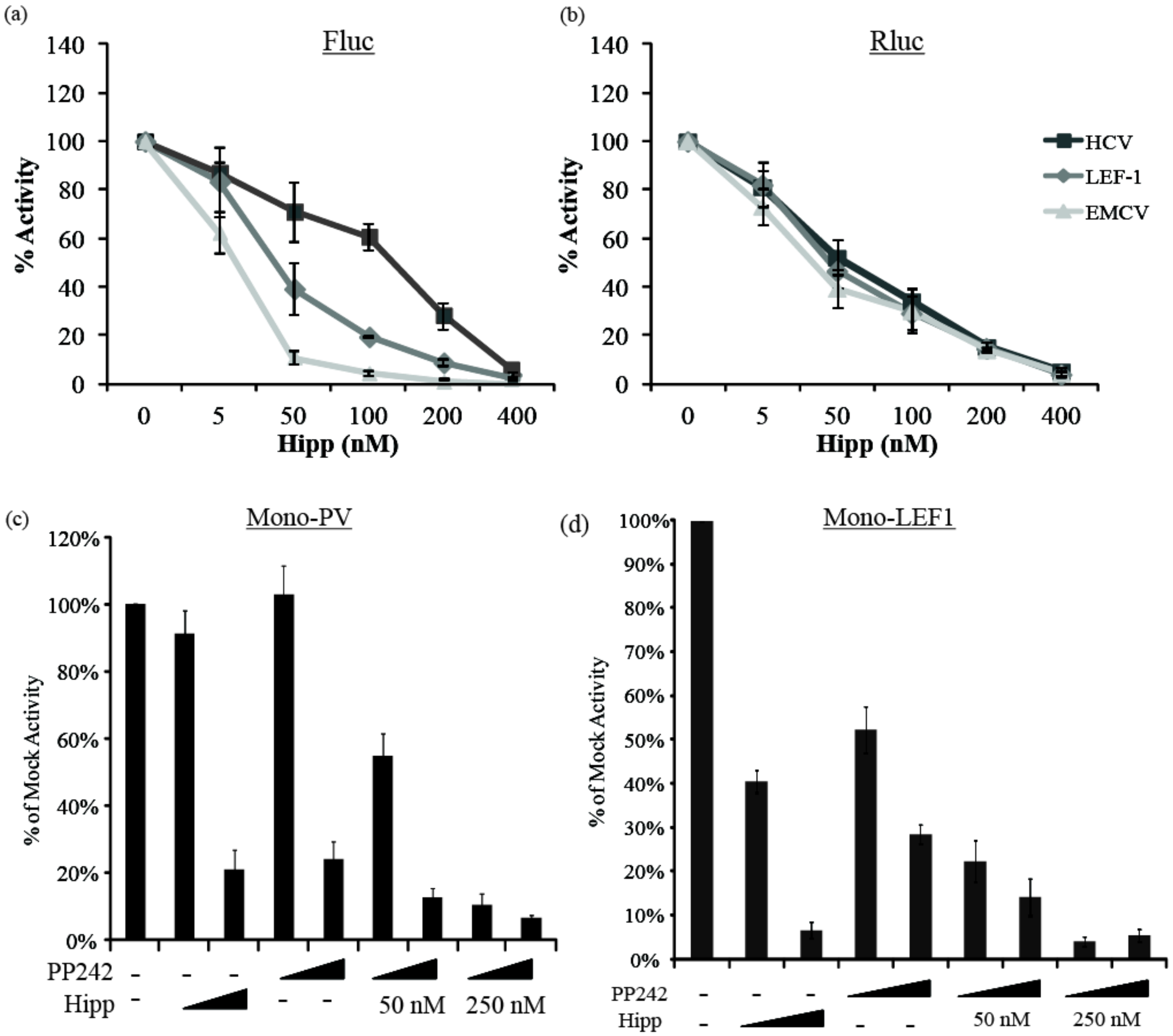

Figure S1. Effect of Hippuristanol and PP242 Treatment on Cellular and Viral IRESes. K562 cells transfected with pRstF-HCV, LEF-1, or EMCV dicistronic vectors. Cells were treated with titrating concentrations of hippuristanol (Hipp) for 24 h. Graph displays percent activity of (a) Renilla (Rluc) or (b) Firefly (Fluc) luciferase after treatment. K562 cells were transfected with (c) mono-LEF1 or (d) mono-PV and β-galactosidase control vectors, treated with hippuristanol, PP242, or both drugs and harvested at 24 h post-treatment for luciferase assay. IRES activity was normalized with β-gal values and represented as a percentage of mock treated cells.

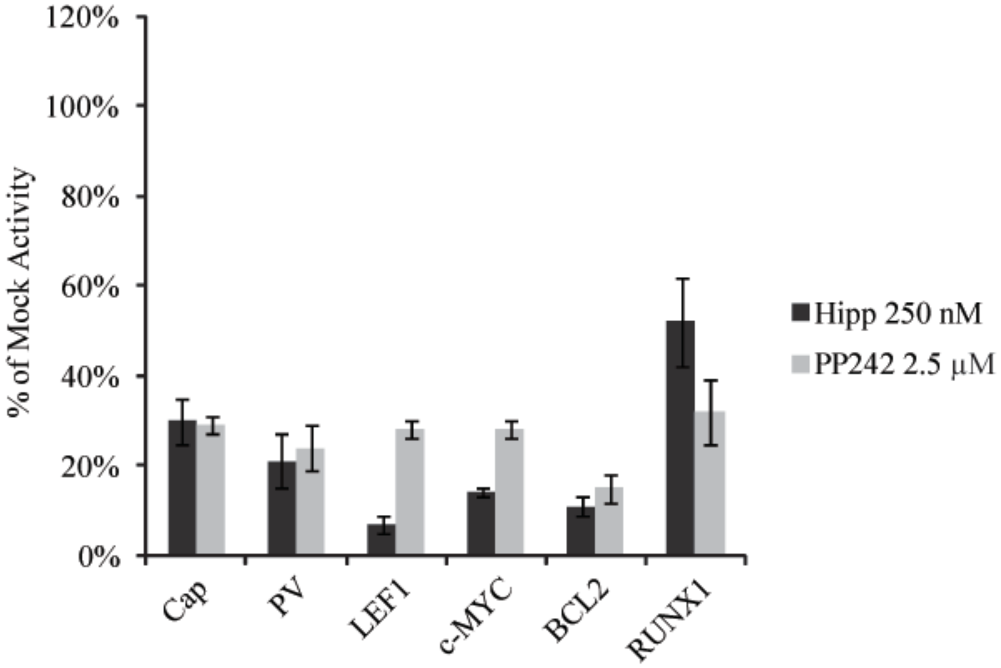

Figure S2. High Doses of Hippuristanol and PP242 Suppress Both IRES and Cap-Mediated Translation Activity. Monocistronic reporters for the cap-translated Firefly luciferase (Cap) the following IRESs: poliovirus (PV), LEF1, c-MYC, BCL2 and RUNX1, were co-transfected with the β-galactosidase control vector into K562 cells. Cells were harvested after 24 h with 250 nM hippuristanol (Hipp) and 2.5 μM of PP242. All raw values were normalied with β-gal and activity is represented as a percentage of the activity of each reporter in mock treated cells.

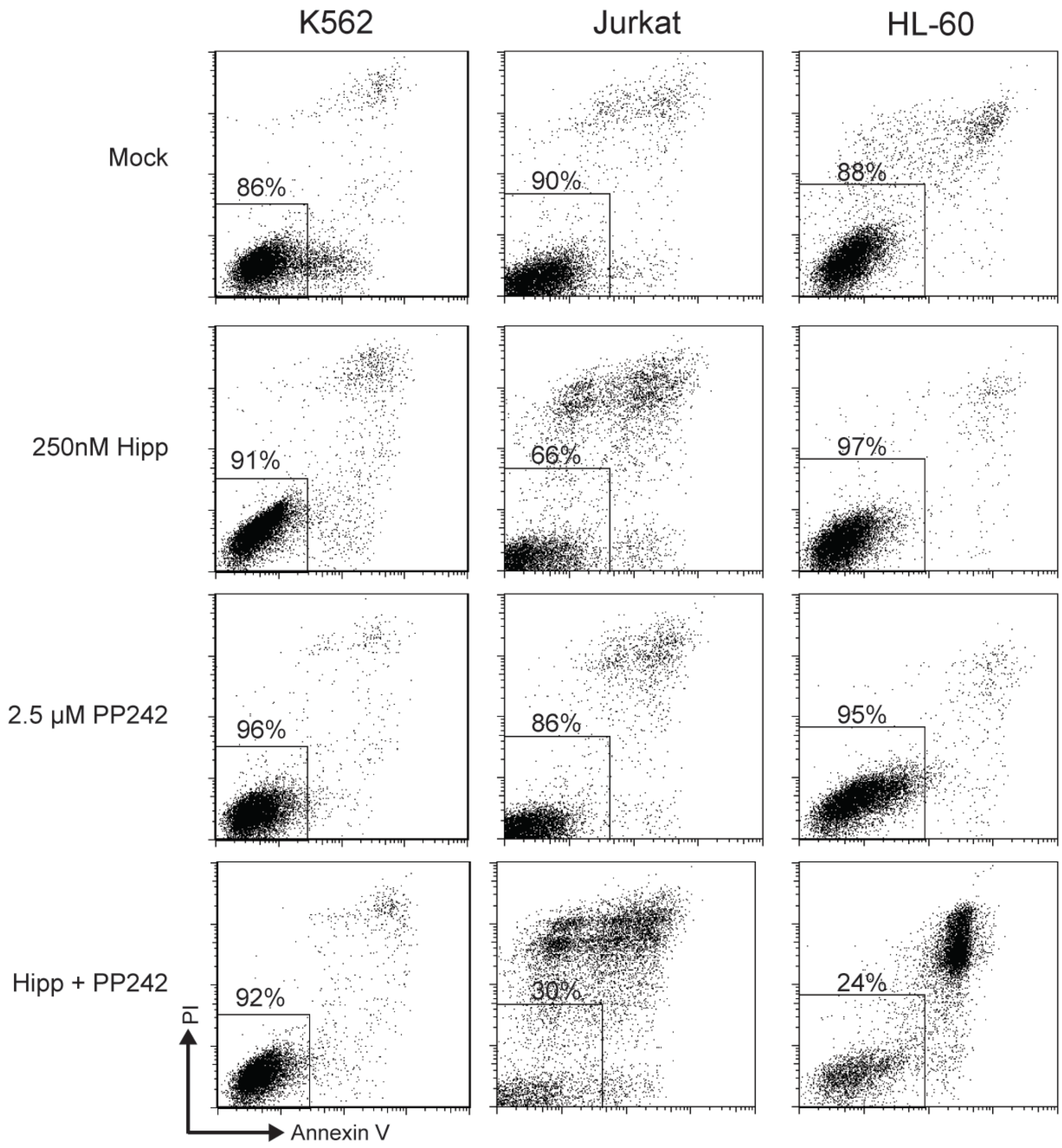

Figure S3. The Effects of Hippuristanol and PP242 Treatment on Leukemia Cell Viability. Apoptosis assays were performed on K562, Jurkat, and HL-60 cells. Cells were treated with hippuristanol (Hipp), PP242 (PP) or both drugs for 48 h, stained with PI and annexin- V and analyzed by flow cytometry. The percent of live cells (annexin-V^-, PI^-) are indicated.

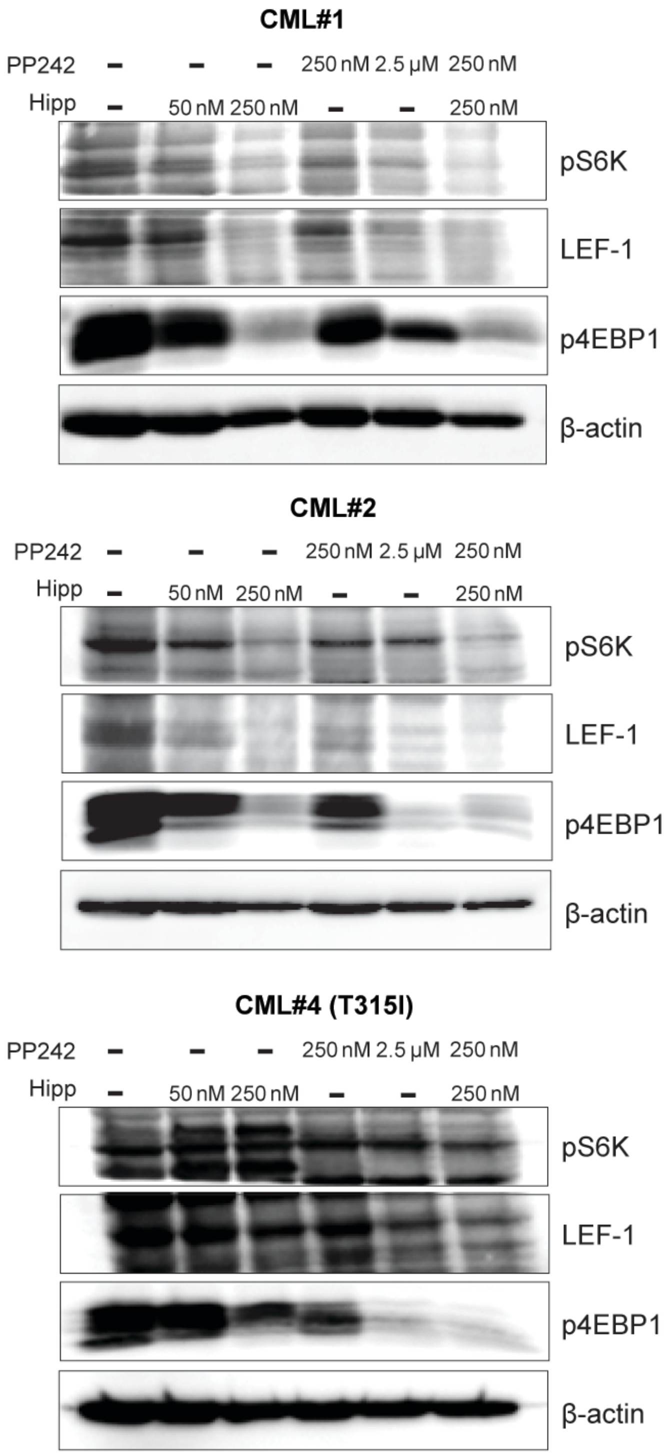

Figure S4. Hippuristanol and PP242 Treatment of Primary CML Patient Samples. Primary blast crisis CML patient cells were treated with hippuristanol, PP242, or both drugs and harvested at 24 h for Western blot analysis. Patients CML#1 and CML#2 displayed reduced LEF-1protein levels with drug treatments compared to CML#4.

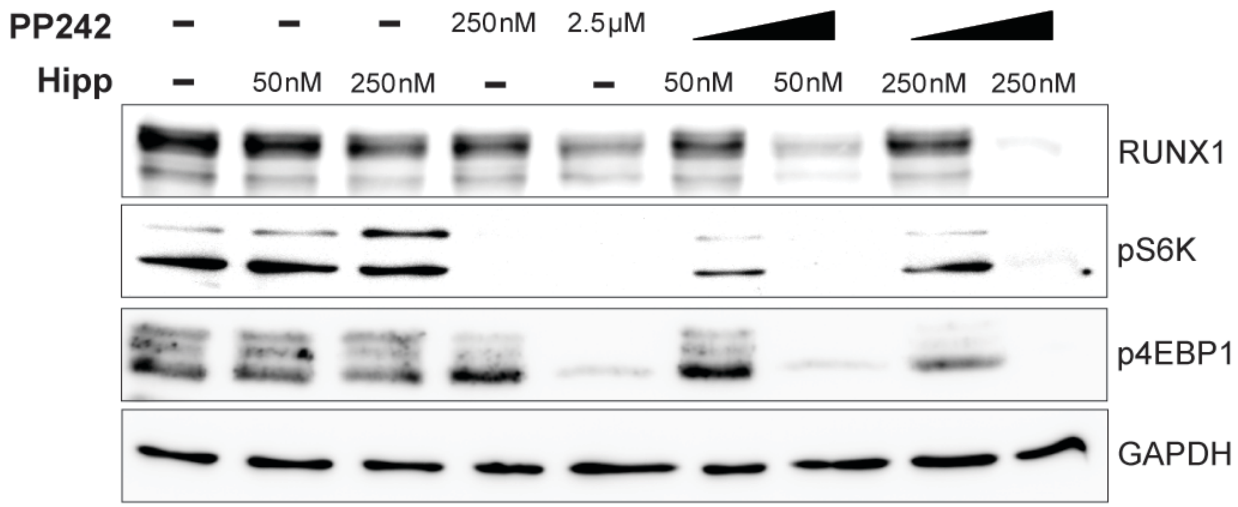

Figure S5. Hippuristanol and PP242 Treatment of AML HL-60 Cells. HL-60 cells were treated with hippuristanol, PP242, or both drugs and harvested at 24 h for Western blot analysis.
